# Vascular Effects of Polyphenols from *Agrimonia eupatoria* L. and Role of Isoquercitrin in Its Vasorelaxant Potential in Human Arteries

**DOI:** 10.3390/ph15050638

**Published:** 2022-05-22

**Authors:** Jéssica Malheiros, Daniela M. Simões, Pedro E. Antunes, Artur Figueirinha, Maria Dulce Cotrim, Diogo A. Fonseca

**Affiliations:** 1 Laboratory of Pharmacology and Pharmaceutical Care, Faculty of Pharmacy, University of Coimbra, 3000-548 Coimbra, Portugal; jessica_051992@hotmail.com (J.M.); daniela.sm.simoes@gmail.com (D.M.S.); mdcotrim@ci.uc.pt (M.D.C.); 2 Coimbra Institute for Clinical and Biomedical Research (iCBR), Faculty of Medicine, University of Coimbra, 3000-548 Coimbra, Portugal; p.engracia.antunes@gmail.com; 3 Center for Innovative Biomedicine and Biotechnology, University of Coimbra, 3000-548 Coimbra, Portugal; 4 Centre of Cardiothoracic Surgery, University Hospital and Faculty of Medicine of Coimbra, 3000-075 Coimbra, Portugal; 5 Clinical Academic Centre of Coimbra, CACC, 3000-075 Coimbra, Portugal; 6 Laboratory of Pharmacognosy, Faculty of Pharmacy, University of Coimbra, 3000-548 Coimbra, Portugal; 7 REQUIMTE/LAQV, R. D. Manuel II, Apartado 55142, 4051-401 Oporto, Portugal

**Keywords:** *Agrimonia eupatoria* L., vasorelaxation, cyclooxygenase, nitric oxide, human internal thoracic artery, vasoprotective potential, polyphenols, isoquercitrin, quercetin

## Abstract

*Agrimonia eupatoria* L. has been traditionally used for the treatment of inflammatory diseases but also as a hypotensive. To our knowledge, only one study has previously suggested an improvement in vascular endothelial function in diabetic conditions, as the underlying mechanisms and responsible compounds are unknown. In this study, we aimed to assess the direct vascular effects of *Agrimonia eupatoria* L. in human arteries. The infusion elicited a mild increase in basal vascular tone and a significant potentiation of the adrenergic contraction of 49.18% at 0.02 mg/mL, suggesting the presence of compounds with mild vasoconstrictor activity. In contrast, the ethyl acetate fraction inhibited adrenergic contraction by 80.65% at 2 mg/mL and elicited no effect on basal vascular tone. A potent concentration-dependent vasorelaxation was observed for both the infusion and the ethyl acetate fraction (maximal relaxation above 76% and 47%, respectively). Inhibition of nitric oxide synthase and cyclooxygenase elicited significant decreases in the vasorelaxation to the infusion, as, for the ethyl acetate fraction, only the cyclooxygenase pathway appeared to be involved. Isoquercitrin elicited a vasoactivity consistent with the ethyl acetate fraction, suggesting this is a major component responsible for the vasorelaxant properties of *A. eupatoria*. Further research is warranted to fully evaluate its vasoprotective properties with therapeutic potential in several conditions, e.g., atherosclerosis.

## 1. Introduction

*Agrimonia eupatoria* L. (*A. eupatoria*) is a plant that belongs to the Rosaceae family and is widely distributed throughout Europe, Asia, North America and Africa [1]. In folk medicine, this plant has been primarily used for the treatment of inflammatory diseases, due to the high content of polyphenols [2,3], but the ethnomedicinal use as hypotensive has also been reported [4]. Moreover, the antidiabetic potential of *A. eupatoria* has been shown, involving several mechanisms from glucose formation and absorption to insulin secretion [5,6].

Regarding its vascular effects, improved acetylcholine-induced vasorelaxation has been shown in isolated aortic rings from diabetic animals treated with the aqueous extract of *A. eupatoria*, compared to untreated diabetic animals but not to healthy controls [6]. As the high content of polyphenols was suggested to play a role in this vascular protection, whether the improved endothelium-dependent vasorelaxation was related to the antidiabetic effects of this extract or to a direct effect on the vasculature remains to be clarified. To our knowledge, no other studies have reported the direct effects of this plant on the vasculature and the underlying mechanisms are unknown, as well as the responsible compounds.

In this context, we aimed to assess the direct vascular effects of *A. eupatoria* in human internal thoracic arteries (ITAs). To this purpose, we tested the vascular activity of an infusion, an ethyl acetate (EtOAc) fraction and the three major constituents of these extracts, i.e., isoquercitrin, tiliroside and *p*-coumaric acid. Furthermore, we evaluated the role of the two major endothelial pathways, i.e., nitric oxide (NO) and cyclooxygenase (COX), in its vascular bioactivity. Here, we showed that the extracts of *A. eupatoria* display potent vasorelaxant activity in human arteries, which is mediated by both NO and COX pathways for the infusion and specifically by COX for the EtOAc fraction. Of the tested compounds, isoquercitrin was found to be the responsible for the vasorelaxant properties of the EtOAc fraction of *A. eupatoria*. Further research is warranted to fully evaluate its vasoprotective properties with therapeutic potential in several conditions, e.g., atherosclerosis.

## 2. Results

### 2.1. Phytochemical Profile of Infusion and EtOAc Fraction

**Figure 1:**
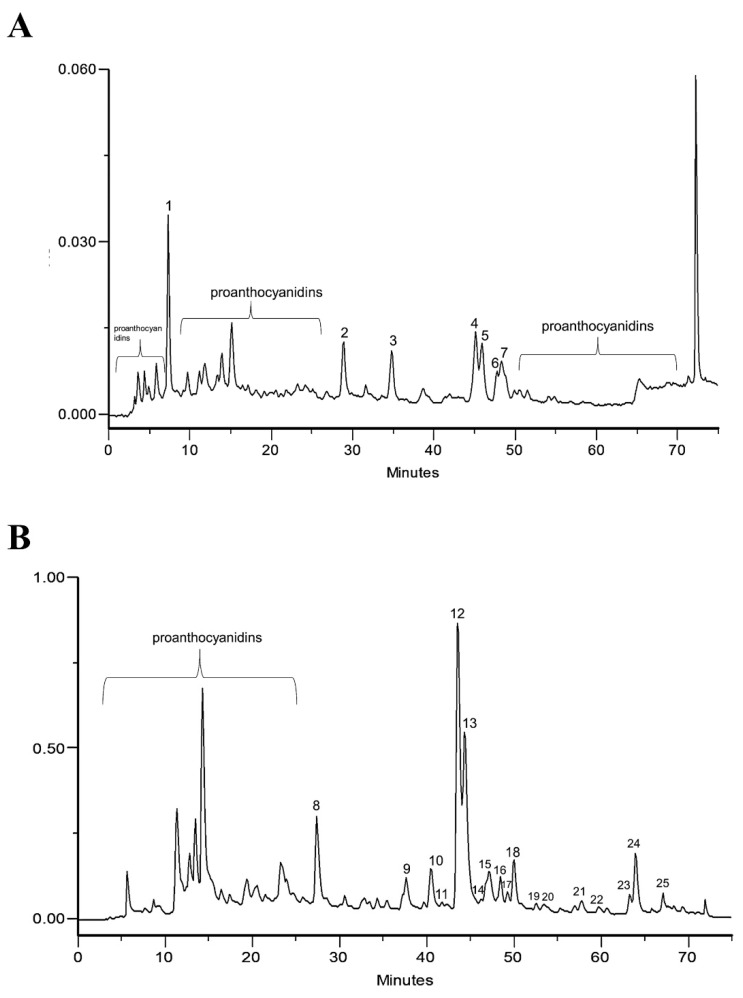
HPLC-PDA profile of *A. eupatoria*: (**A**) infusion and (**B**) ethyl acetate fraction. Both chromatograms were recorded at 280 nm.

The phenolic profile of the infusion and EtOAc fraction of *A. eupatoria* was determined through HPLC-PDA (Figure 1A,B, respectively).

Regarding the infusion, the UV spectrum of peak 1 suggests the presence of a benzoic acid derivative (wavelength maximum near 313 nm). Peak 2 with wavelength maxima at 310 nm was identified as *p*-coumaric acid, using the commercial standard compound. Peaks 3, 4 and 5 were 3-*O*-glycosylated quercetin derivatives, as suggested by their UV spectra profile, with band I near 350 nm, with absorptivity lower than band II with wavelength maxima near 250 and shoulder at approximately 265 and 295 nm [7]. Peak 4 was identified as isoquercitrin (quercetin 3-*O*-glucoside) through a comparison of retention time with a commercial standard. Peaks 3 and 5 were previously identified by using HPLC-PDA-ESI/MSn, respectively, as quercetin *O*-galloyl-hexoside and quercetin *O*-malonyl-hexoside [3]. In peak 6, UV spectra, retention time and the wavelength maxima near 265 and 346 nm with a shoulder near 314 nm suggested the presence of kaempferol *O*-*p*-coumaroyl-glucoside (tiliroside), as confirmed with a commercial standard. Peak 7 exhibited a UV spectra profile with wavelength maxima of 248 and 371 nm with a shoulder 322 nm, which is characteristic of ellagic acid, also confirmed with the standard compound. Proanthocyanidins were detected due to their characteristic spectral profiles and UV maxima (near 246 and 278 nm) [8]. The complete characterization of mixture components was previously carried out and published in Santos et al. [3].

In the EtOAc chromatogram (Figure 1B), peak 8 was identified as *p*-coumaric acid. Peaks 9 and 11 with wavelength maxima at 272 and 335 was characteristic of apigenin derivatives [9]. Peak 11 was identified as isovitexin with a commercial standard. Peaks 10, 12, 13, 14 and 17 exhibited UV profile spectra characteristic of quercetin derivatives [7]. Specifically, peak 12 was identified as isoquercitrin, also reported in the infusion (peak 4). Peaks 10 and 13 suggest the presence of quercetin *O*-galloyl-hexoside and quercetin *O*-malonyl-hexoside, respectively. These compounds were previously reported [3] and were identified by HPLC-PDA-ESI/MSn; additionally, these compounds were also present in the infusion (peaks 3 and 5). Peak 15 was identified as ellagic acid, also identified in the infusion (peak 7). Peaks 16, 21 and 25 showed UV spectra, retention time and wavelength maxima near 265 and 346 nm with a shoulder at 314 nm. This behavior is characteristic of kaempferol derivatives [7]. Peak 16 was identified as tiliroside. This compound was also identified in the infusion (peak 6). Peaks 18, 19 and 20 were also identified as kaempferol derivatives due to the UV profile and wavelength maxima at 264 nm, 346 nm and a shoulder at 296 nm [7]. Finally, peak 19 was identified as kaempferol-*O*-malonyl-hexoside by HPLC-PDA-ESI/MSn [3].

Based on the chromatograms, *p*-coumaric acid, tiliroside and quercetin derivatives were identified as the major constituents in the infusion and the EtOAc fraction and were therefore quantified by HPLC (Table 1). As can be seen, quercetin derivatives were the major class of compounds in both extracts.

### 2.2. Vascular Effects

#### 2.2.1. Infusion

**Figure 2:**
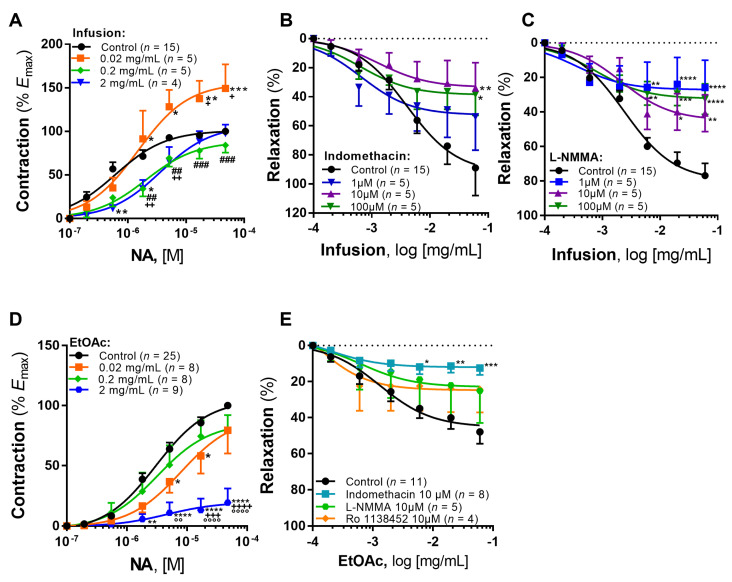
Vascular activity of the infusion (**A**–**C**) and EtOAc fraction (**D**–**E**) of *A. eupatoria*. Infusion: (**A**) Effect on the noradrenaline-induced contraction; * *p* < 0.05 vs. control, ** *p* < 0.01 vs. control, *** *p* < 0.001 vs. control, ^##^ *p*< 0.01 vs. 0.2 mg/mL, ^###^ *p* < 0.001 vs. 2 mg/mL, ^+^ *p* < 0.05 vs. 2 mg/mL, ^++^ *p* < 0.01 vs. 2 mg/mL. (**B**) Influence of COX blocking with indomethacin on the vasorelaxation to the infusion; * *p* < 0.5 vs. control, ** *p* < 0.01 vs. control. (**C**) Influence of endothelial nitric oxide synthase blocking with L-NMMA on the vasorelaxation to the infusion; * *p* < 0.05 vs. control, ** *p* < 0.01 vs. control, *** *p* < 0.001 vs. control, **** *p* < 0.0001 vs. control. Significance refers to unpaired two-way analysis of variance (ANOVA) with Tukey’s multiple comparisons test. EtOAc fraction: (**D**) Effect on the noradrenaline-induced contraction; * *p* < 0.05 vs. control, ** *p* < 0.01 vs. control, **** *p* < 0.0001 vs. control, ^+++^ *p* < 0.001 vs. 2 mg/mL, ^++++^ *p* < 0.0001 vs. 2 mg/mL, °° *p* < 0.01 vs. 2 mg/mL, °°°° *p* < 0.0001 vs. 2 mg/mL. (**E**) Influence of COX blocking with indomethacin, endothelial nitric oxide synthase blocking with L-NMMA and blocking PGI_2_ receptors with a selective antagonist Ro 1138452 on the vasorelaxation to EtOAc fraction; * *p* < 0.05 vs. control, ** *p* < 0.01 vs. control, *** *p* < 0.001 vs. control.

The infusion of *A. eupatoria* induced a mild increase in basal vascular tone (*E*_max_ = 1.10 ± 0.67 mN; pEC_50_ = 2.72 ± 0.51, *p* < 0.05 vs. vehicle). Furthermore, the lower concentration of infusion (0.02 mg/mL) elicited a significant potentiation of the noradrenaline-induced contraction (Figure 2A and Table 2), with an *E*_max_ increase of 49.18% vs. control (*p* < 0.001, Figure 2A). This response was also significantly higher compared to the other infusion concentrations (0.2 and 2 mg/mL), which only lead to a significant decrease in potency in comparison with control: 0.2 mg/mL (*p* < 0.05) and 2 mg/mL (*p* < 0.001).

Next, we assessed the vasorelaxant activity of the infusion and the role of the two major endothelial pathways of vasorelaxation, i.e., COX and NO. As can be seen, the infusion induced a marked concentration-dependent vasorelaxation (*R*_max_ of 88.99 ± 18.89% and 76.88 ± 6.98%, respectively in Figure 2B,C). Incubation with indomethacin (COX inhibitor) produced a significant decrease in the maximal vasorelaxation to the infusion (*p* < 0.01 10 µM vs. control and *p* < 0.05 100 µM vs. control, Figure 2B). Incubation with L-NMMA (NO synthase inhibitor) elicited significant decreases in maximal vasorelaxation in all tested concentrations (Figure 2C).

#### 2.2.2. EtOAc Fraction

EtOAc fraction did not induce any change in basal vascular tone (*E*_max_ = 0.12 ± 0.32 mN; pEC_50_ = 2.67 ± 3.69, *p* < 0.05 vs. infusion). Furthermore, the lower concentration (0.02 mg/mL) changed the noradrenaline-induced contraction only in intermediate concentrations of noradrenaline (statistically significant decreases of 27.13% and 27.72%, *p* < 0.05 in Figure 2D), even though no significant differences were detected in efficacy or potency vs. control (Table 3). Furthermore, the higher concentration of the EtOAc fraction (2 mg/mL) elicited a significant decrease of 80.65% in the *E*_max_ in the noradrenaline cumulative concentration-response curves (CCRCs) (Figure 2D), thus suggesting a decrease in efficacy.

The EtOAc fraction elicited a vasorelaxant effect (*R*_max_ = 47.97 ± 6.55%, Figure 2E), which was completely abolished by endothelial denudation (*R*_max_ = −25.37 ± 39.05%; data not shown in graph), thus suggesting this effect is endothelium-dependent. Similarly, the role of NO and COX pathways was also evaluated. Incubation with 10 μM L-NMMA produced a non-significant decrease in the vasorelaxation to the EtOAc fraction (*R*_max_ = 25.14 ± 17.80%, Figure 2E). In contrast, incubation with 10 μM indomethacin decreased significantly the vasorelaxation (*R*_max_ = 12.45 ± 3.78%, *p* < 0.001 vs. control, Figure 2E).

Given this significant decrease of the relaxation with the incubation with indomethacin, we hypothesized that the COX-derived prostanoid, prostacyclin, could be responsible for this vasorelaxant effect. However, the incubation with the IP receptor selective antagonist (Ro 1138452) produced a nonsignificant decrease of the maximal relaxation to the EtOAc fraction (*R*_max_ = 24.33 ± 12.75%, Figure 2E).

#### 2.2.3. Isoquercitrin, Tiliroside and *p*-Coumaric Acid

The marked vasorelaxant effect previously observed lead to the investigation of the compounds that could be responsible for this action. Hence, the quantification of the major compounds identified in the infusion and the EtOAc fraction was carried out (Table 1). Based on this quantification, we extrapolated the amount of compound present in the higher concentration of the EtOAc fraction used in the vascular studies described above (i.e., 2 mg/mL). Thereafter, we used the following concentrations: isoquercitrin (28 µg/mL), tiliroside (3 µg/mL) and *p*-coumaric acid (1.5 µg/mL). Given the predominant composition in quercetin derivatives (Table 1), we also included quercetin as a reference compound for comparison of vascular effects. 

**Figure 3:**
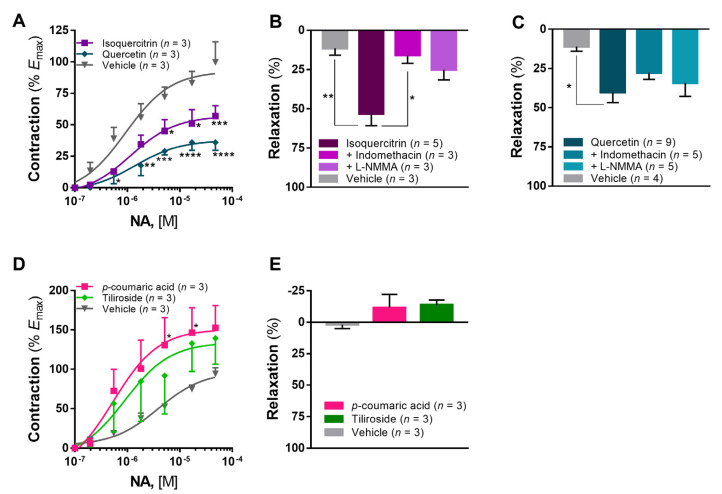
Vascular activity of isoquercitrin, quercetin, tiliroside and *p*-coumaric acid. (**A**) Effect of isoquercitrin and quercetin (28 µg/mL) on the contraction induced by noradrenaline. Vasorelaxant effect on isoquercitrin (**B**) and quercetin (**C**) and influence of COX and endothelial nitric oxide synthase blocking with indomethacin (10 µM) and L-NMMA (10 µM), respectively. (**D**) Effect of *p*-coumaric acid (1.5 µg/mL) and tiliroside (3 µg/mL) on the noradrenaline-induced contraction. (**E**) Vasorelaxant effect of *p*-coumaric acid and tiliroside. * *p* < 0.05, ** *p* < 0.01, *** *p* < 0.001, **** *p* < 0.0001 vs. vehicle.

Isoquercitrin elicited a statistically significant decrease of 42.86% (*p* < 0.001 vs. vehicle) in the maximal contraction to noradrenaline (*E*_max_ = 56.94 ± 8.28%, Figure 3A) and a potent vasorelaxant activity with *R*_max_ of 53.29 ± 7.42 (*p* < 0.01 vs. vehicle, Figure 3B). As seen, incubation with indomethacin decreased significantly the vasorelaxation elicited by isoquercitrin (*R*_max_ = 15.94 ± 5.10%, *p* < 0.05 vs. isoquercitrin, Figure 3B), whereas incubation with L-NMMA led to a nonsignificant reduction (*R*_max_ = 25.16 ± 6.45%, Figure 3B). In the same concentration, quercetin was able to elicit a more pronounced inhibition of noradrenaline-induced contraction (*E*_max_ = 35.94 ± 6.25%, *p* < 0.0001 vs. vehicle, Figure 3A). Moreover, quercetin elicited a marked vasorelaxant effect (*R*_max_ = 40.21 ± 6.49, *p* < 0.05 vs. vehicle, Figure 3C), which was not significantly different from isoquercitrin (*p* > 0.05). Differently from isoquercitrin, the vasorelaxant effect of quercetin was not affected by preincubation with indomethacin (*R*_max_ = 27.74 ± 4.18%) or L-NMMA (*R*_max_ = 34.12 ± 8.64%).

Both *p*-coumaric acid and tiliroside elicited a potentiation of the noradrenaline contraction (*E*_max_ of 152.80 ± 28.23% and 139.40 ± 33.10%, respectively, Figure 3D), even though only *p*-coumaric acid produced statistically significant changes in intermediate concentrations (*p* < 0.05 vs. vehicle). In the tested concentrations, these compounds did not elicit any vasorelaxation in the ITA (*R*_max_ of −11.43 ± 10.69% and −13.69% ± 3.93%, respectively, Figure 3E).

## 3. Discussion

To our knowledge, only one report [6] has focused on the vascular activity of *A. eupatoria*, showing that treatment with the aqueous extract improved acetylcholine-induced vasorelaxation in aortic rings isolated from diabetic animals. Importantly, this improvement was observed only in comparison with untreated diabetic animals but not with healthy controls. Whether such effect was a result from the anti-diabetic properties of this extract or from a direct vascular activity remained to be clarified.

Here, we tested the direct vascular activity of *A. eupatoria* and showed that its infusion exhibits potent vasorelaxant activity in human arteries (Figure 2). As a low concentration (0.02 mg/mL) elicited a potentiation of the maximal response to noradrenaline, the infusion displayed a mixed vascular effect, thus suggesting some compounds may elicit an increase in vascular tone and others a decrease. Both endothelial pathways (i.e., NO and COX) seem to be involved (Figure 2). Interestingly, the magnitude of the vasorelaxation (above 76%) could also suggest a direct vascular smooth muscle relaxation (i.e., endothelium-independent), which remains to be confirmed.

A potent vasorelaxant effect was also observed with the EtOAc fraction (Figure 2), although it was lower compared to the infusion (above 47% and 76%, respectively). In contrast to the observed with the infusion, the EtOAc fraction elicited a decrease in noradrenaline-induced contraction at the higher concentration (2 mg/mL). Furthermore, the COX endothelial pathway was found to be the underlying mechanism for the vasorelaxation of the EtOAc fraction. This difference in vasorelaxant activity, particularly NO-mediated, must be highlighted, as it may result from the extraction process, with a loss of compounds such as procyanidin B2. In fact, endothelium-dependent vasorelaxation to procyanidin B2 has been described in the ITA [10], which involves NO synthesis and secretion by endothelial cells and partially of prostacyclin and also activation of BK_Ca_ and K_ATP_ channels, as well as K_V_ and IK_Ca_. Moreover, procyanidins have also been reported to stimulate the production of prostacyclin in the ITA [11]. Although we did not identify procyanidin B2 in the extracts that were assayed in our work, the EtOAc fraction was found to be rich in procyanidin dimers in a previous report [3]. In fact, this compound has been identified in the EtOAc fraction of the same batch of this plant species [3,12].

Based on the findings, we hypothesized that prostacyclin, which is a fundamental endothelium-derived relaxing factor that increases cAMP leading to vasorelaxation, could be the mediator of this response. However, experiments with the IP receptor antagonist Ro 1138452 did not confirm our hypothesis (Figure 2E). Beyond prostacyclin, other vasodilator metabolites have been described, such as epoxyeicosatrienoic acids or EETs (namely 14,15-EET and 11,12-EET), cytochrome P450 metabolites and prostaglandin D2 and E2 also via COX [13]. Previous evidence suggests that 11,12-EET elicits relaxation in ITA through the activation of BK_Ca_ channels and the vanilloid transient receptor potential channel 4 and canonical transient receptor potential channel 1 in smooth muscle cells [14,15]. As to prostaglandins D2 and E2, previous reports have shown no vasorelaxation in the ITA, rather an EP receptor-mediated vasoconstriction in response to prostaglandin E2 [16,17]. Overall, the precise mechanism through which *A. eupatoria* elicits vasorelaxation remains to be fully characterized.

In terms of composition, both extracts displayed a high phenolic content and quercetin derivatives were found to be the predominant compounds, namely isoquercitrin, together with tiliroside and *p*-coumaric acid (Table 1). Therefore, we hypothesized that these compounds could be responsible for the observed vasorelaxant activity. Based on our findings (Figure 3), isoquercitrin showed vasoactive properties consistent with the EtOAc fraction (i.e., ability to reduce noradrenaline-induced contraction and potent COX-mediated vasorelaxant activity), thus suggesting this is the compound responsible for such activity. This is a compound known for multiple activities, such as antiviral against human herpes viruses [18], antioxidant, anti-inflammatory and anticancer effects, among others [19].

In regard to its cardiovascular activity, previous studies have highlighted the antihypertensive potential of isoquercitrin through an inhibitory effect on angiotensin converting enzyme [20]. Also, it has been shown to elicit K^+^ channel- and endothelial NO-mediated vasodilation in perfused rat mesenteric arteries [21]. Moreover, isoquercitrin displays antiapoptotic activity in vascular endothelial cells, which may be relevant in atherogenesis [22,23]. Atheroprotective properties have also been attributed to enzymatically modified isoquercitrin or EMIQ (i.e., isoquercitrin with malto-oligosaccharides) in atherogenic ApoE-deficient mice [24]. In clinical studies, Bondonno et al. [25] showed no acute changes in blood pressure or brachial NO-mediated endothelium-dependent relaxation in healthy individuals. More recently, EMIQ was shown to acutely improve brachial flow-mediated dilatation in patients at risk for cardiovascular disease [26].

Since our extract and the EtOAc fraction contained a high content of quercetin derivatives, namely, isoquercitrin, we aimed to assess the vascular activity of quercetin as a reference compound in human arteries. In our study, quercetin decreased significantly the maximal contractile response to noradrenaline (Figure 3A) and elicited a potent vasorelaxation (Figure 3C). To our knowledge, this is the first report on the vasoactivity of quercetin in human arteries, as the observed effects are generally consistent with previous reports on rat aorta [27,28], rat basilar artery [29] and rat tail main artery [30]. Furthermore, indomethacin and L-NMMA did not change significantly the vasorelaxation to quercetin, suggesting that the endothelium is not involved in this vasorelaxant effect in the ITA. In rat aorta, a previous study showed that the vasorelaxant effect of quercetin is partially dependent on the endothelium nitric oxide synthase and endothelium-derived hyperpolarizing factor [28]. More recently, this vasorelaxation partially mediated by NO was confirmed, even though endothelium nitric oxide synthase blocking with L-NAME decreased only the potency and not the maximal relaxation [29]. A similar finding was described for COX blocking with indomethacin. Other studies have suggested endothelium-independent relaxation with the involvement of calcium and potassium channels [30,31]. Overall, our findings are consistent with this evidence suggesting the involvement of endothelium-independent mechanisms.

In regard to the other tested compounds, *p*-coumaric acid and tiliroside, these elicited no vasorelaxation and no statistically significant effects on noradrenaline-induced maximal contraction at the tested concentrations. In the literature, *p*-coumaric acid has been shown to protect against doxorubicin-induced oxidative stress in the rat heart through free radical scavenging properties [32], as well as to display antiplatelet activity [33], but no report has emerged on its vascular activity.

As to tiliroside, its antihypertensive and vasorelaxant effects have been reported [34]. In that study, the authors reported a dose-dependent decrease in blood pressure in hypertensive rats, a concentration-dependent vasodilation of rat mesenteric arteries, as well as a blockage in the membrane depolarization-induced increase of intracellular Ca^2+^ concentration and decreased voltage-activated peak amplitude of the L-type Ca^2+^ channel current in VSMCs. This antihypertensive potential has been recently confirmed [35], as tiliroside antagonized the transient hypertension evoked by the administration of angiotensin II in a dose-dependent way.

The absence of vasorelaxant effect in our assays, particularly for tiliroside, could be explained by the low concentrations that were used. However, our focus was to assess the compounds responsible for the vasorelaxation to *A. eupatoria*; thus, we extrapolated the concentrations of compounds that were present in the higher concentration of the EtOAc fraction that was used (2 mg/mL), rather than use a range of concentrations or higher concentrations as described in those previous reports.

Some limitations should be considered. First, the variability in the obtained data may derive from the use of human arterial tissue harvested from patients with underlying cardiovascular conditions and therefore a diverse biological background. However, this may also constitute an advantage, as it provides a better translation of the findings to humans and especially when considering patients with vascular disease. Second, we did not test if direct smooth muscle relaxation is involved in the observed vasorelaxant effects. However, it is unlikely that it is a major mechanism, considering the results obtained after endothelium removal or in the presence of inhibitors of endothelial pathways.

To our knowledge, this is the first report on the direct vascular effects of extracts and compounds from *A. eupatoria*. Collectively, the data demonstrates the vasorelaxant potential of its infusion and EtOAc fraction. Both the COX and NO pathways appear to be involved in the activity of the infusion of *A. eupatoria*, as the COX pathway was found to be the major pathway involved in the effects elicited by the EtOAc fraction and specifically isoquercitrin, even though the specific mediators remain unclear.

## 4. Materials and Methods

### 4.1. Plant Material

Aerial parts of *A. eupatoria* were provided by Segredo da Planta, Portugal (batch number 5870 and quality control data identifying no defects), and a voucher specimen (T. Batista 02009) has been deposited at the Herbarium of Medicinal and Aromatic Plants, Faculty of Pharmacy, University of Coimbra. The method used for obtaining the infusion of *A. eupatoria* from the aerial parts has been previously published [3]. Briefly, 20 g of pulverized plant was infused into 600 mL of water for 15 min. After this procedure, the infusion was vacuum-filtered and washed with *n*-hexane (1:1) to eliminate fat-soluble compounds.

The EtOAc fraction was obtained from half of the crude extract, which was fractionated by repeated extraction with ethyl acetate (3 × 225 mL), also according to previous published data [3]. After the extraction, the sample was concentrated with water in a rotavapor at 30 °C and then freeze-dried.

### 4.2. HPLC-PDA

Phytochemical characterization of the infusion and EtOAc fraction was carried out using HPLC coupled with a photodiode array (PDA) detector (Gilson Electronics SA, Villiers le Bel, France). Data were treated with Unipoint^®^ 2.10 (Gilson, Middleton, WI, USA). The samples (100 μL) were injected in a Waters^®^ Spherisorb S5 ODS-2 column (250 × 4.6 mm i.d., 5 μm), protected with a guard cartridge C18 (30 × 4 mm i.d., 5 μm) (Nucleosil, Macherey-Nagel, Düren, Germany), and eluted at a flow rate of 1 mL/min and 35 °C. The mobile phase consisted of 5% formic acid (*v*/*v*) (eluent A) and methanol (eluent B). The following gradient was utilized: 0–60 min (5–50% B), 60–70 min (50–100% B) and 70–75 min (100–100% B). The concentration for injection was 1 mg/mL for both samples. The chromatographic profiles were recorded at 280 nm.

Quantification was carried out using peak area in the chromatograms obtained by HPLC-PDA against external standards at appropriate wavelengths: 315 nm for *p*-coumaric acid and tiliroside and 256 nm for quercetin derivatives. Three independent injections were performed for each sample, injecting 100 μL of extract and standards dissolved in water, except quercetin, which was dissolved in 1:1 methanol in water. The identification of compounds was performed by comparing retention time and their UV spectra with previous analysis [3] and commercial reference compounds. Detection (LOD) and quantification limits (LOQ) were determined from the parameters of the calibration curves represented in Table 4.

The identification of infusion and EtOAc fraction compounds was performed by comparing retention times and their UV spectra with *p*-coumaric acid, isovitexin, isoquercitrin (quercetin-3-*O*-glucoside), ellagic acid and tiliroside and data previously published by other authors.

### 4.3. Vascular Activity Studies

Experiments were performed on distal segments of ITA harvested from patients undergoing coronary revascularization, with the approval by the Ethics Committee of Faculty of Medicine of University of Coimbra (CE-118/2018) and of Coimbra University Hospitals (reference PC-388/08) and following informed consent. Vascular tissue preparation was carried out as described in [36].

The effect on basal vascular tone was assessed by CCRCs to the infusion or the EtOAc fraction (0.002–0.2 mg/mL) or vehicle, and the results were expressed as absolute contraction (in millinewton, mN).

The vasorelaxant effect was tested with CCRCs (0.02–0.2 mg/mL) to the infusion, EtOAc fraction or vehicle, after sustained pre-contraction with noradrenaline (20 µM), as results were expressed as a percentage of the maximum contraction to NA (%).

The modulatory effect on the noradrenaline-induced contraction was assessed with CCRCs to noradrenaline (0.1–48 µM) before and after 30-min pre-incubations with the infusion or EtOAc fraction (0.02, 0.2 and 2 mg/mL) or vehicle, as the results were expressed as the percentage of the maximal contraction in the control curve, i.e., absence of infusion (% *E*_max_).

The influence of NO and COX pathways was assessed with *N*^G^-monomethyl-L-arginine (L-NMMA) and indomethacin (1, 10 and 100 µM), respectively. For the infusion, concentrations of 1, 10 and 100 µM were used, as only the 10 µM concentration was used in experiments with the EtOAc fraction. The role of the endothelium on the vasorelaxant effect was also assessed by mechanical endothelium removal (which was confirmed with the absence of a relaxant response to acetylcholine). Furthermore, the influence of the prostacyclin receptor (IP receptor) was evaluated with the selective antagonist Ro 1138452 (10 µM).

In these experimental setups, the vehicle (distilled water) did not elicit any changes (data not shown).

Thereafter, the effects of compounds were assessed in the same conditions described above for noradrenaline-induced contraction and the influence of NO and COX with L-NMMA and indomethacin (10 µM, respectively) in the vasorelaxant effect. The concentrations that were assayed were based on compound quantification as described above, *p*-coumaric acid (1.5 µg/mL), quercetin and isoquercitrin (28 µg/mL), and tiliroside (3 µg/mL). For these experiments, vehicle refers to distilled water (for tiliroside and *p*-coumaric acid) or DMSO in water (for isoquercitrin and quercetin).

Tissue viability was tested with potassium chloride (KCl, 60 mM) at the beginning and at the end of all experiments.

### 4.4. Analysis of Results

Data from vascular activity studies were generally expressed as mean ± standard error of mean (SEM) unless specified otherwise, and *n* indicates the number of experiments. In assays in which the modulatory effect on the noradrenaline-induced contraction was tested, potency was expressed as the negative logarithm of the effective concentration (in mol/L or M) of noradrenaline able to induce half of the maximum contraction (pEC_50_, -log [M]). In vasorelaxation assays, potency was expressed as the negative logarithm of the effective concentration (mg/mL) of the infusion or the EtOAc fraction able to reduce noradrenaline precontraction to half of its maximum, i.e., relaxation of 50% (pIC_50_, -log[mg/mL]). Efficacy was expressed either as %*E*_max_ (maximal contraction, %) or %*R*_max_ (maximal relaxation, %), respectively, for those assays.

Statistical analysis was performed using GraphPad Prism 7^®^ (GraphPad Inc., La Jolla, CA, USA). First, the normality of data was checked through a Shapiro–Wilk test. Data from CCRCs were analyzed by two-way ANOVA with Tukey’s multiple comparisons test to identify differences in specific concentrations throughout the curves. Parameters of potency from CCRCs and data on vascular effects of compounds were analyzed by unpaired one-way ANOVA with Tukey’s multiple comparisons. *p* < 0.05 was considered to indicate a statistically significant difference.

### 4.5. Reagents

Chemicals used for Krebs–Henseleit buffer preparation and arterial ring viability testing in the pharmacological studies were purchased from Sigma-Aldrich^®^ (St. Louis, MO, USA). The following commercial standards were used in the ITA assays: quercetin 3-*O*-glucoside (Sigma, 17793-10MG-F), quercetin (G Buchs SG. K148-/49/2), *p*-coumaric acid (Sigma, C9008) and tiliroside (Extrasynthese, 1001 S, 20316-62-5). The selective prostacyclin IP receptor antagonist Ro 1138452 hydrochloride (4268) was purchased from Tocris (Bristol, UK).

Chemicals used for phytochemical characterization were purchased from Merck^®^ (Darmstadt, Germany) and correspond to the highest grade commercially available. The reference compounds used were: ellagic acid (Sigma, E2250-5G), *p*-coumaric acid (Sigma, C-9008, Lot: 22H0312), quercetin (G Buchs SG., Buchs, Switzerland, K148-/49/2), quercetin-3-*O*-glucoside (Sigma, 17793-10MG-F), tiliroside (Extrasynthese, Genay, France, 1001 S, Lot: 12080209), vitexin (Extrasynthese, 1232 S) and isovitexin (Extrasynthese, 1235 S).

## 5. Conclusions

To our knowledge, this is the first report on the direct vascular effects of extracts and compounds from *A. eupatoria*. In our study, we demonstrated that an infusion of *A. eupatoria* may exhibit a mixed vascular activity, in which some compounds may promote mild vasoconstriction while others elicit pronounced vasorelaxation. Interestingly, the EtOAc fraction elicited a decrease in the contraction to noradrenaline, while maintaining a marked vasorelaxant effect. In our study, we showed also that this remarkable vasorelaxation involves both the COX and the NO endothelial pathways, while the COX pathway exhibits a central role in the vasorelaxant effect of the EtOAc fraction. Isoquercitrin showed a marked COX-mediated vasorelaxation, and this result corroborates the activity observed for the EtOAc fraction. Overall, these findings suggest *A. eupatoria* exhibits vasoprotective properties and warrant further research to clarify the mechanistic basis for this vascular activity (i.e., identify the specific targets and mediators) and to validate the therapeutic potential in several conditions, e.g., atherosclerosis.

## Figures and Tables

**Table 1:** Quantification of *p*-coumaric acid, quercetin derivatives and tiliroside in the infusion and the EtOAc fraction of *A. eupatoria* by HPLC-PDA and the respective concentrations in organ bath experiments.

	Compound (Tentative Identification)	μg of Compound per 100 g of Extract	μg of Compound per 2 mg of Extract/mL ^1^
**Infusion**			
Peak 2	*p*-coumaric acid	73.00	1.470
Peak 3	Quercetin derivatives ^2^	150.7	3.140
Peak 4	Isoquercitrin	1500	30.16
Peak 5	Quercetin derivatives ^2^	270.8	5.550
Peak 6	Tiliroside	147.0	2.940
**EtOAc fraction**			
Peak 8	*p*-coumaric acid	70.00	1.400
Peak 10	Quercetin derivatives ^2^	157.9	3.150
Peak 12	Isoquercitrin	1400	28.00
Peak 13	Quercetin derivatives ^2^	333.5	6.670
Peak 16	Tiliroside	138.0	2.760

^1^ Major concentration of the extract of *A. eupatoria* used in vascular activity experiments. ^2^ Results expressed as quercetin equivalent.

**Table 2:** Pharmacological parameters from vascular activity studies with *A. eupatoria* infusion.

Studies (Incubation with Infusion or Compound)	Concentration	Maximal Effect ^1^	Potency ^2^	*n*
Influence on adrenergic contraction (infusion)	Control	100.00 ± 0.00	6.21 ± 0.07	15
	0.02 mg/mL	149.18 ± 27.76 ***	5.83 ± 0.18	5
	0.2 mg/mL	84.08 ± 8.55 ^###^	5.69 ± 0.14 *	5
	2 mg/mL	97.65 ± 10.15 ^+^	5.44 ± 0.14 ***	4
Role of COX (indomethacin)	Control	89.99 ± 18.89	2.40 ± 0.17	15
	1 μM	53.77 ± 22.91	3.18 ± 0.36	5
	10 μM	34.03 ± 17.36 **	2.89 ± 0.44	5
	100 μM	38.44 ± 14.18 *	3.19 ± 0.29	5
Role of NO (L-NMMA)	Control	76.88 ± 6.98	2.62 ± 0.09	15
	1 μM	26.00 ± 15.91 ****	3.45 ± 0.56	5
	10 μM	40.73 ± 10.62 **	2.67 ± 0.24	5
	100 μM	32.51 ± 8.7 ****	3.12 ± 0.22	5

Results presented as mean ± SEM and *n* corresponds to the number of experiments. ^1^ Maximal effect expressed as maximal contraction (%*E*_max_ to noradrenaline) for adrenergic contraction studies or maximal relaxation for studies on the role of COX and NO in vasorelaxation (%*R*_max_). ^2^ Potency expressed as pEC_50_ for incubation studies or as pIC_50_ for vasorelaxation studies. * *p* < 0.5 vs. control, ** *p* < 0.01 vs. control, *** *p* < 0.001 vs. control, **** *p* < 0.0001 vs. control, ^###^ *p* < 0.001 vs. 0.02 mg/mL, ^+^ *p* < 0.05 vs. 0.02 mg/mL.

**Table 3:** Pharmacological parameters from vascular activity studies with the EtOAc fraction of *A. eupatoria*.

Studies (Incubation with Fraction or Compound)	Concentration	Maximal Effect ^1^	Potency ^2^	*n*
Influence on adrenergic contraction (EtOAc fraction)	Control	100.00 ± 0.00	5.51 ± 0.06	25
	0.02 mg/mL	79.43 ± 19.32 ^++++^	5.09 ± 0.26	8
	0.2 mg/mL	78.40 ± 13.63 ^°°°°^	5.49 ± 0.27	8
	2 mg/mL	19.35 ± 11.82 ****	5.30 ± 0.73	9
Role of mediators in vasorelaxation	Control	47.95 ± 6.55	2.88 ± 0.22	11
Indomethacin	10 μM	12.45 ± 3.78 ***	3.75 ± 0.76	8
L-NMMA	10 μM	25.14 ± 17.80	3.19 ± 1.25	5
Ro 1138452	10 μM	24.33 ± 12.75	4.00 ± 1.51	4

Results presented as mean ± SEM, and *n* corresponds to the number of experiments. ^1^ Maximal effect expressed as maximal contraction (%*E*_max_ to noradrenaline) for adrenergic contraction studies or maximal relaxation for studies on the role of COX and NO in vasorelaxation (%*R*_max_). ^2^ Potency expressed as pEC_50_ for incubation studies or as pIC_50_ for vasorelaxation studies. *** *p* < 0.001 vs. control, **** *p* < 0.0001 vs. control, ^++++^ *p* < 0.0001 vs. 2 mg/mL, °°°° *p* < 0.0001 vs. 2 mg/mL.

**Table 4:** Linearity, limit of detection (LOD) and limit of quantification (LOQ) of the three standards compounds used as reference.

Standard Compound	Range Concentrations (μg/mL)	*n* ^1^	Slope	Intercept	R^2^	LOD (μg/mL)	LOQ (μg/mL)
*p*-coumaric acid	2.5–15	6	8.95 × 10^6^	3.90 × 10^6^	0.9960	0.49 ± 0.32	2.66 ± 0.21
Quercetin	2.5–125	5	3.10 × 10^6^	2.62 × 10^6^	0.9932	8.18 ± 3.66	29.26 ± 3.26
Tiliroside	5–25	6	3.65 × 10^6^	1.11 × 10^6^	0.9979	1.30 ± 0.36	3.65 ± 0.31

^1^ Number of points used for the regression of standard solutions. Injections were done in duplicate.

## Data Availability

The data presented in this study are available on request from the corresponding author. The data are not publicly available in order to fully preserve patient confidentiality, as defined in the project approved by the Ethics Committee of the Faculty of Medicine of the University of Coimbra (CE-118/2018). In addition to the corresponding author, data requests may also be addressed to the Centre of Cardiothoracic Surgery of the University Hospital of Coimbra, Praceta Mota Pinto, 3000-075 Coimbra, Portugal.
